# ICT1 Promotes Osteosarcoma Cell Proliferation and Inhibits Apoptosis via STAT3/BCL-2 Pathway

**DOI:** 10.1155/2021/8971728

**Published:** 2021-01-22

**Authors:** Xiaohui Pan, Jingxue Tan, Xiaokun Weng, Rui Du, Yuqing Jiang, Yiping Weng, Dong Zhou, Yifei Shen

**Affiliations:** ^1^Department of Orthopedics, The Affiliated Hospital of Nanjing Medical University, Changzhou No.2 People's Hospital, Changzhou, Jiangsu 213003, China; ^2^Department of Radiotherapy, The Affiliated Hospital of Nanjing Medical University, Changzhou No.2 People's Hospital, Changzhou, Jiangsu 213003, China; ^3^Department of Foot and Ankle Surgery, Binzhou Medical University Hospital, Binzhou, 256600 Shandong Province, China; ^4^Department of Orthopedics, Shanghai Tenth People's Hospital, School of Medicine, Shanghai Tongji University, 200120 Shanghai, China

## Abstract

Osteosarcoma (OS) is a familiar malignant bone tumor that occurs mainly in adolescents. Immature colon carcinoma transcript-1 (ICT1) is an important member of the large mitoribosomal subunit in mitochondrial ribosomes, which has been shown to be closely related to tumorigenesis. Its expression and function in OS, however, remained unclear. Here, we showed that ICT1 was significantly upregulated in OS and promoted the growth of OS cells. Mechanistically, ICT1 acted as an oncogene in OS and promoted proliferation and inhibited apoptosis of OS cells through the STAT3/BCL-2 axis. These results reveal a novel insight into the role of the ICT1/STAT3/BCL-2 axis in OS and therefore may represent a novel molecular target for novel treatments.

## 1. Background

Osteosarcoma (OS) is a common bone cancer in teenagers and originates in interstitial cells [1]. The basic characteristics of OS are its heterogeneity, genomic instability, and the high risk of metastasis, primarily to the lungs [2]. Currently, surgery, chemotherapy, immuno-therapy, and gene therapy represent the main treatments [3]. However, the strong proliferative and migratory capacity of OS has led to poor prognosis [4]. Therefore, it is important for us to investigate novel oncogenes that play crucial roles in the origin and progression of OS, in order to develop better treatments for patients with this devastating disease.

Immature colon carcinoma transcript-1 (ICT1) belongs to a family of four putative mitochondrial translation release factors and controls the terminal stage of translation [5]. Furthermore, studies have revealed that ICT1 plays a significant role in the development and progression of tumors. Evidence suggests that ICT1 is closely associated with proliferation in some human cancer cells, such as hepatocellular carcinoma, gastric cancer, non-small-cell lung cancer, and breast cancer [6, 7]. However, the effect of ICT1 in OS is unclear.

Proteins of the B-Cell CLL/Lymphoma 2 (BCL-2) family play a crucial role in the control of apoptosis and have proapoptotic and antiapoptotic activities. Some proteins from this family, such as BCL-2, are antiapoptotic, whereas others such as BAX are proapoptotic. The BCL-2 protein plays an important role in the control of prolonged cell survival, and as mentioned, it is a major antiapoptotic protein [8]. BCL-2 is responsible for preventing the mitochondrial release of cytochrome c, which leads to caspase-3 cleavage and cell apoptosis [9, 10].

STAT3 is a critical signal protein that is involved in a variety of growth factors and cytokines, which triggers a variety of biological outcomes, including cell growth, differentiation, and survival [11]. Moreover, STAT3 is reported to be an important oncogene in many malignant tumors. It is reported that activation of STAT3 was involved in the development and progression of gastric cancer [12]. Previous work demonstrated that STAT3 signaling was activated by RHPN2 to promote malignant cell behaviors in ovarian cancer [13]. Li et al. reported that Farnesoid X receptor inhibited the progression of colorectal cancer by suppressing JAK2/STAT3 signaling [14]. However, the role of STAT3 signaling in OS is not entirely clear.

In this study, the expression level of ICT1 in OS cells was found to be higher than that in osteoblast cells. Database analysis showed that patients with the high expression of ICT1 had a significantly poorer prognosis. Our data showed that the expression of ICT1 increased in OS. The biological functions of ICT1 in the growth of OS were investigated *in vitro* and *in vivo*. Furthermore, we identified a novel mechanism showing that ICT1 promoted OS cell proliferation and inhibited apoptosis through the STAT3/BCL-2 axis. Thus, ICT1 may serve as an anticancer biomarker with high efficacy.

## 2. Materials and Methods

### 2.1. Cell Culture and Reagents

Two human OS cell lines (MNNG-HOS, 143B) and human osteoblast cells (hFOB1.19) were purchased from the Cell Bank of the Chinese Academy of Sciences (Shanghai, China). The American Type Culture Collection (ATCC, Manassas, VA, USA) provided the human OS U-2OS cell line. All cell lines were cultured under standard conditions. Human OS cells were cultured at 37°C in a 5% CO_2_ atmosphere, and the hFOB1.19 cells were cultured in a 5% CO_2_ atmosphere at 34.5°C. Antibodies against ICT1 (AP20382b; Abgent, San Diego, CA, USA), cleaved caspase-3 (YT6161, Immunoway, Beijing, China), cleaved caspase-9 (YP0598, Immunoway, Beijing, China), BCL-2 (ab32124; Abcam, Cambridge, UK), *β*-actin (M1210-2; Hua'an Biology, Chuzhou, China), STAT3 (#4904, Cell Signaling Technology, Danvers, MA, USA), and p-STAT3(#9145, Cell Signaling Technology, Danvers, MA, USA) were used.

### 2.2. Establishment of Stable ICT1 Knockdown Cell Lines and BCL-2 Overexpression Cell Lines

The stable ICT1 knockdown and BCL-2 overexpressing cell lines (U-2OS and 143B) were established using plasmids containing sh-ICT1 or BCL-2 and a negative control plasmid provided by OBiO Technology (Shanghai, China). The shRNA sequences for ICT1 are as follows: 5′-GCTGTTAATGCTTGTCTATAACTCGAGTTATAGACAAGCATTAACAGC-3′ and 5′-GCAGAATGTGAACAAAGTGAACTCGAGTTCACTTTGTTCACATTCTG C-3′. The overexpression RNA sequence for BCL-2 was 5′-GTTGTAGCGGGACAC CTACTGAAAGTTCTCTTCAGTAGGTGTCCCGCTACAAAAAAACTTA-3′. HEK 293T cells provided by OBiO Technology were used to package these plasmids into virus particles, and then, the viral titers were measured. To produce stable ICT1-knockdown cells and BCL-2 overexpressing cells, the target cells were infected with 1 × 10^8^ lentivirus-transducing units with 6 mg/mL polybrene (Sigma-Aldrich, St. Louis, MO, USA). After 72 h, infected cells were screened with 2.5 mg/mL of puromycin. Finally, we used western blotting and qRT-PCR to measure the efficiency.

### 2.3. RNA Isolation and qRT-PCR Analysis

Total RNAs were extracted and reverse transcribed as detailed previously [15]. ICT1 and BCL-2 transcripts were quantified with a SYBR Green qPCR Master Mix (Roche, Switzerland). The primer sequences used for measuring the expression level of ICT1 were as follows: forward 5′-CAGCCTGGACAAGCTCTACC-3′, reverse: 5′-GGAACCTGACTTCTGCCTTG-3′. The reaction conditions were set up in accordance with the manufacturer's instructions. *β*-Actin and 18S were used as internal controls.

### 2.4. Western Blotting

Total cellular protein was extracted using a protein extraction buffer (Beyotime, Shanghai, China). Proteins were resolved by sodium dodecyl sulfate-polyacrylamide gel electrophoresis and transferred onto a nitrocellulose membrane (Millipore, Belfor, MA, USA). After blocking, the membranes were incubated with primary antibodies at 4°C overnight. The next day, the membranes were probed with secondary antibodies. Finally, protein bands were visualized using a chemiluminescence substrate (Share-bio, Shanghai, China).

### 2.5. Immunohistochemistry (IHC)

Alena Biotechnology Co., Ltd. (Xi'an, China) provided a microarray containing tissue from 40 OS patients, and the IHC assay was carried out as previously described [16]. We employed the corresponding primary antibodies at a 1 : 200 dilution to detect Ki67. We took micrographs of all the sections using a fluorescence microscope (Carl Zeiss, Oberkochen, Germany). The final IHC score was counted by multiplying the intensity score with the quantity score as previously described [17]. These scores were judged independently by two experienced pathologists in a blinded manner.

### 2.6. Cell Counting Kit-8 (CCK-8) Assay and Colony Formation Assay

The CCK-8 assay was performed according to the vendor's instructions (Dojindo Molecular Technologies, Japan). Briefly, 3 × 10^3^ cells were seeded into 96-well plates, and absorbance at a wavelength of 450 nm was measured at 0, 24, 48, 72, 96, and 120 h using a tablet reader (Thermo Fisher Scientific, Waltham, MA, USA).

The infected OS cells were cultured in a six-well plate at an initial cell density of 1 × 10^3^ cells/well. After two weeks, ice-cold PBS was used to wash the colonies. Then, cell pellets were fixed with 4% paraformaldehyde and stained with 0.1% crystal violet. These were then photographed, and the cell numbers were counted.

### 2.7. Cell Apoptosis

In order to assess cell apoptosis, adherent cells were separated after culturing for 24 h in serum-free medium. Cells were then stained with the Annexin V/FITC Kit (BD Biosciences, San Jose, CA, USA) following the manufacturer's instructions. Finally, flow cytometry was used to assess the results.

### 2.8. Caspase-3/9 Activity Assay

Caspase-3 and caspase-9 activity was measured by using a caspase-3 and caspase-9 activity assay kit according to manufacturer's protocol (Beyotime Institute of Biotechnology, Chuzhou, China). At 48 h posttransfection, the OS cells (U-2OS and 143B) were harvested and lysed at 4°C for 30 min using lysis buffer. Total protein was incubated with 10 *μ*L Ac-DEVD-pNA and 10 *μ*L Ac-LEHD-pNA at 37°C for 2 h. The absorbance of each well was measured with a microplate reader set at 405 nm.

### 2.9. Xenograft Tumor Production in Nude Mice

Balb/c nude mice (no sex limitation; 20–25 g) were fed and housed and experimented on at the East China Normal University and were carried out according to the animal experimental protocols authorized by the Animal Care and Use Committee of the Animal Care and Use Committee of the Affiliated Hospital of Nanjing Medical University, Changzhou No.2 People's Hospital. The mice were assigned to several groups with five mice per group. In brief, the Balb/c nude mice were anesthetized with 1.5% pentobarbital sodium. Then, U-2OS cells (1.5 × 10^6^) were subcutaneously inoculated into mice. Tumor size data were collected every five days, and all of the mice were euthanized using CO_2_ after 20 days. All tumors were removed, and their weight and size were recorded. There are other novel methods to measure 3D tumor volume [18].

### 2.10. Terminal Deoxynucleotidyl Transferase (TdT) dUTP Nick-End Labeling (TUNEL) Assay

The percentage of apoptotic cells in the xenografted tumors was quantified using a TUNEL kit (Roche, Basel, Switzerland). This assay was carried out as previously reported [17].

### 2.11. Statistical Analyses

The GraphPad software was used for statistical analyses. The expression of all data was represented as mean ± SD. Comparisons between different groups were performed using Student's *t*-test, and a value of *p* < 0.05 was considered to be statistically different.

## 3. Results

### 3.1. ICT1 Is Upregulated in OS Cell Lines and Promotes Proliferation and Inhibits Apoptosis of OS Cells *In Vitro*

**Figure 1:**
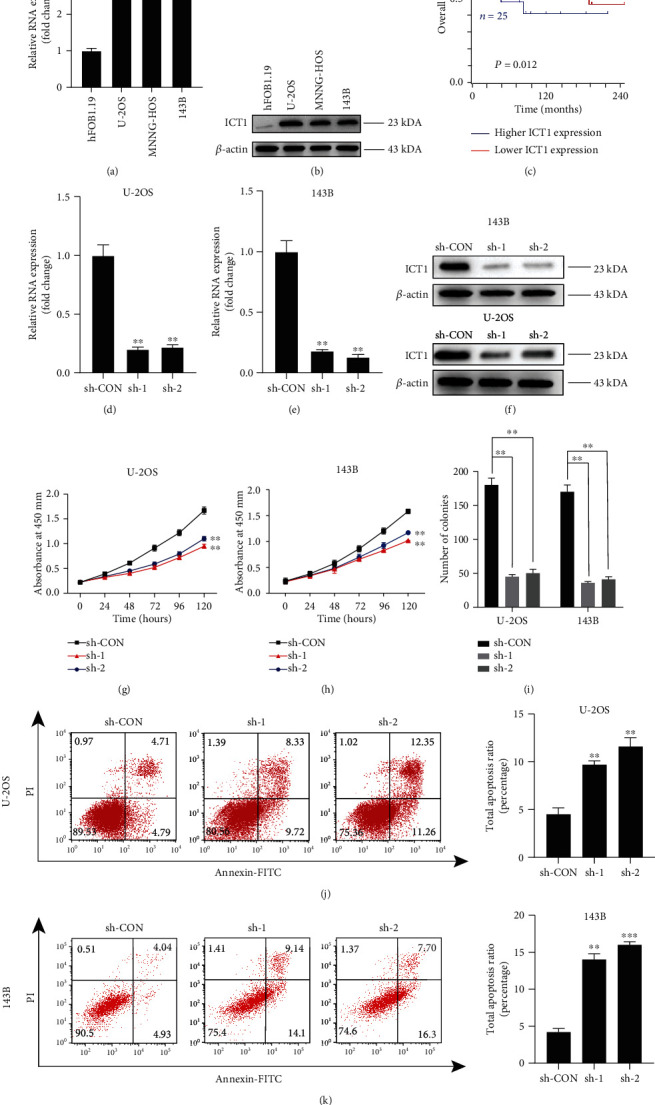
The expression of ICT1 is closely related to the overall survival, and ICT1 promotes proliferation and inhibits apoptosis of OS cells *in vitro.* (a) The mRNA expression patterns of ICT1 in normal osteoblast cell line (hFOB1.19) and OS cell lines (U-2OS, MNNG-HOS, and 143B) by qPCR. Values are means ± SD, ^∗∗^*p* < 0.01, ^∗∗∗^*p* < 0.001 (Student's *t*-test). (b) The expression patterns of ICT1 in normal osteoblast cell line (hFOB1.19) and OS cell lines (U-2OS, MNNG-HOS, and 143B) by western blotting. (c) Kaplan–Meier analysis of overall survival rate related to the expression of ICT1 in 88 OS cases based on a human osteosarcoma gene expression database (https://hgserver1.amc.nl/cgi-bin/r2/main.cgi). (d, e) Knockdown efficacy of ICT1 in wild OS cells (U-2OS and 143B) was determined by qPCR. Values are means ± SD, ^∗∗^*p* < 0.01 (Student's *t*-test). (f) Knockdown efficacy of ICT1 in wild OS cells (U-2OS and 143B) was determined by western blotting. (g, h) Knockdown of ICT1 inhibited U-2OS and 143B cell proliferation using the cell counting kit (CCK)-8 assay. Values are means ± SD, ^∗∗^*p* < 0.01 (Student's *t*-test). (i) Knockdown of ICT1 suppressed OS cell (U-2OS and 143B) proliferation using colony formation assay. Values are means ± SD, ^∗∗^*p* < 0.01 (Student's *t*-test). (j, k) Knockdown of ICT1 significantly induced U-2OS and 143B cell apoptosis. Values are means ± SD, ^∗∗^*p* < 0.01 (Student's *t*-test).

First, qRT-PCR analysis and western blotting were used to detect the expression levels of ICT1 in hFOB1.19 cells, a common osteoblast cell line, and in the human OS cell lines (MNNG-HOS, 143B, and U-2OS). The results showed that ICT1 was upregulated in the OS cell lines when compared to the hFOB1.19 cell line (Figures 1(a) and 1(b)). The prognostic value of measuring ICT1 in patients with OS was also revealed by using the following website http://hgserver1.amc.nl, which is an online gene expression database. As shown in Figure 1(c), a Kaplan Meier analysis showed that patients with the high expression of ICT1 had a significantly poorer prognosis (*p* = 0.012). To investigate the role of ICT1 in OS, ICT1 stable knockdown cell lines (U-2OS and 143B cells) were constructed. The knockdown efficiency was validated by qRT-PCR and western blotting (Figures 1(d)–1(f)). We found that the proliferation of OS cells decreased when the expression of ICT1 was silenced, as determined by a CCK8 assay and a colony forming assay (Figures 1(g)–1(i)). However, ICT1 knockdown had little effect on the growth of the normal human osteoblast cell line (hFOB1.19) (Supplementary Fig. 1A-B). The results of cell apoptosis experiments revealed that apoptosis increased when ICT1 was silenced in the OS cells (Figures 1(j) and 1(k)). The above data showed that ICT1 is upregulated in OS cell lines and promotes growth of OS cells *in vitro*.

### 3.2. ICT1 Regulates BCL-2 by Controlling STAT3 Phosphorylation

**Figure 2:**
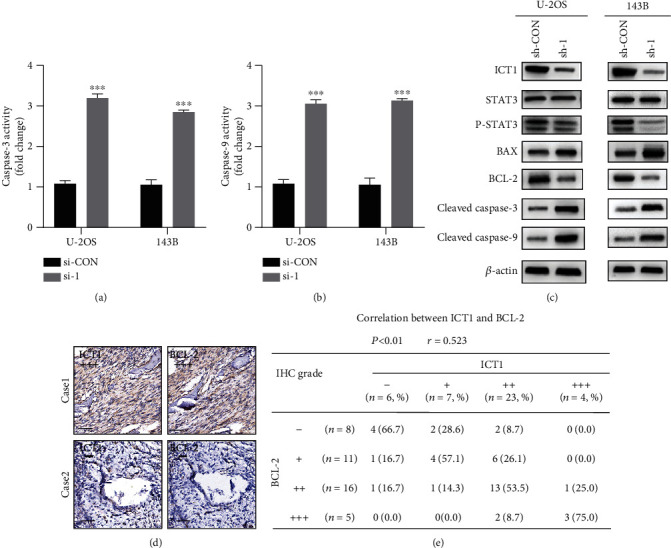
ICT1 regulated BCL-2 through controlling STAT3 phosphorylation. (a, b) Caspase-3 and caspase-9 activity was dramatically increased in the sh-ICT1 OS cells (U-2OS and 143B). Values are means ± SD, ^∗∗^*p* < 0.01 (Student's *t*-test). (c) The expression levels of STAT3, p-STAT3, BAX, BCL-2, cleaved caspase-3, and cleaved caspase-9 were detected using western blotting in sh-Control and sh-ICT1 OS cells (U-2OS and 143B). (d) IHC analysis showed representative positive (up) and negative (down) staining of ICT1 and BCL-2 in consecutive sections; scale bar: 50 *μ*m. (e) Statistical analysis of the correlation between ICT1 and BCL-2 expression in human osteosarcoma tissue microarrays.

Since the cell apoptosis assay indicated that knockdown of ICT1 promoted apoptosis of OS cells, we measured the activities of caspase-3/9. As shown in Figures 2(a) and 2(b), in the U-2OS and 143B cell lines, caspase-3/9 activities were markedly increased after silencing of ICT1. Then, using western blotting, we found that cleaved caspase-3 and cleaved caspase-9 were increased in the U-2OS and 143B cell lines (Figure 2(c)). Moreover, we found that the expression of BCL-2 was markedly reduced, and the expression of BAX was remarkably increased after the silencing of ICT1 (Figure 2(c)). Notably, we found ICT1 expression levels in OS tissues had a positive correlation with the expression levels of BCL-2 (*r* = 0.523, *p* < 0.001) when interrogating the tissue microarray using immunohistochemical analysis (Figures 2(d)–2(e)). The above results indicated that BCL-2 was a potential target for ICT1. STAT3 is an important signaling molecule, and it is closely associated with apoptosis in cancer. Furthermore, the previous studies showed that STAT3 can regulate cell survival by inducing BCL-2 to suppress apoptosis in OS and other tumors [19–22]. Western blotting was used to investigate whether ICT1 regulates BCL-2 through activation of STAT3. The results showed that the knockdown of ICT1 dramatically decreased the level of phosphorylated STAT3 (p-STAT3, Figure 2(c)). These results showed that ICT1 may regulate BCL-2 through the activation of STAT3.

### 3.3. The Effect of ICT1 Knockdown on OS Cell Proliferation and Apoptosis Is Reversed in Part, by BCL-2 Overexpression

**Figure 3:**
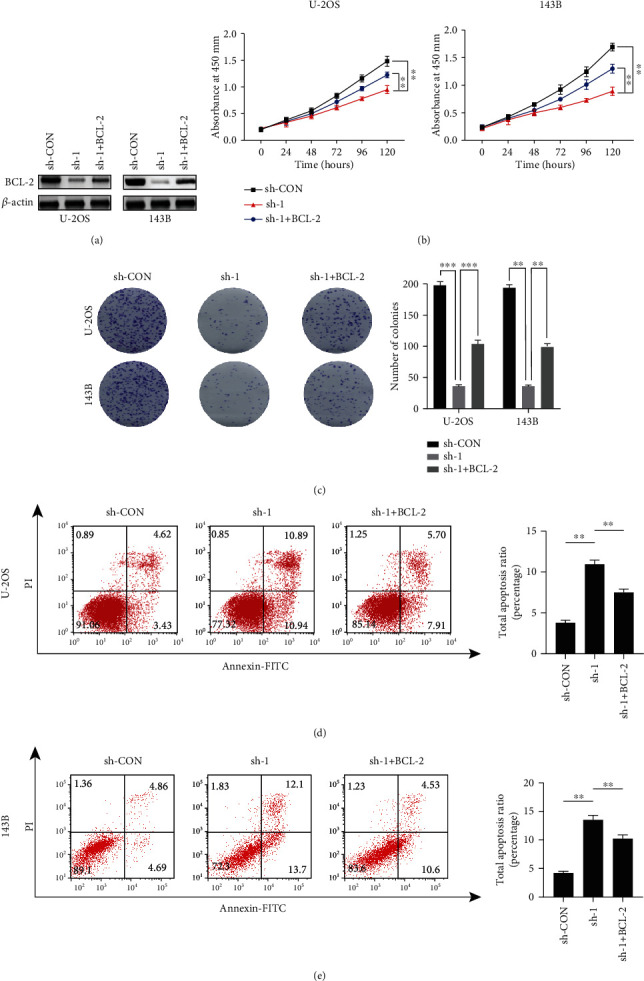
The effect of ICT1 knockdown on osteosarcoma cell proliferation was reversed partly by BCL-2 overexpression. (a) Overexpression efficacy of BCL-2 in sh-ICT1 OS cells (U-2OS and 143B) was detected by western blotting. (b) Overexpression of BCL-2 partly reversed the suppressed effects of ICT1-knockdown on the CCK8 assay of U-2OS and 143B cells. Values are means ± SD, ^∗∗^*p* < 0.01 (Student's *t*-test). (c) Overexpression of BCL-2 partly reversed the suppressed effects of ICT1-knockdown on the colony formation capability of U-2OS and 143B cells. Values are means ± SD, ^∗∗^*p* < 0.01, ^∗∗∗^*p* < 0.001 (Student's *t*-test). (d, e) Overexpression of BCL-2 partly reversed the induce effect of ICT1-knockdown on the apoptosis of OS cells (U-2OS and 143B). Values are means ± SD, ^∗∗^*p* < 0.01 (Student's *t*-test).

In order to determine whether ICT1 regulated cell growth by targeting BCL-2, we firstly overexpressed BCL-2 in stable ICT1 knockdown cell lines (U-2OS cells and 143B cells), and the efficiency of overexpression was detected by western blotting (Figure 3(a)). Then, the CCK8 assays, colony formation assays, and cell apoptosis assays revealed that the overexpression of BCL-2 partly reversed the prohibitive functions of ICT1 knockdown on the protumorigenic features of U-2OS cells and 143B cells *in vitro* (Figures 3(b)–3(e)). The above data suggested that ICT1 promoted growth of OS cells by targeting BCL-2.

### 3.4. ICT1 Promoted OS Cell Growth Targeting BCL-2 *In Vivo*

**Figure 4:**
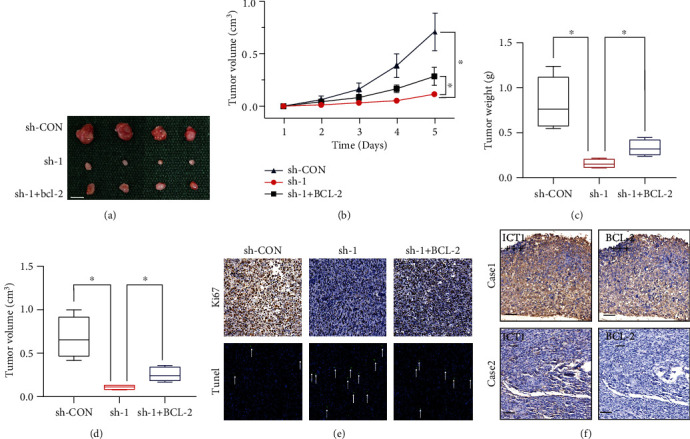
ICT1 promoted OS cell growth targeting BCL-2 *in vivo.* (a) Morphologic characteristics of xenograft tumors from U-2OS/sh-Control group, U-2OS/sh-ICT1 group, and U-2OS/sh-ICT1 + overexpression of BCL-2 group (*n* = 4). Scale bars = 1 cm. (b–d) Overexpression of BCL-2 partly rescued the inhibitory effects of ICT1-knockdown on the growth of U-2OS cells *in vivo*. The volumes and weights of tumors were measured every 5 days. Values are means ± SD, ^∗^*p* < 0.05 (Student's *t*-test). (e) Representative images of Ki67 and TUNEL staining in the xenograft tumors from the sh-Control, sh-ICT1, and sh-ICT1 + overexpression of BCL-2 mice. A TUNEL positive cell is indicated (arrow). (f) IHC analysis showed representative positive (up) and negative (down) staining of ICT1 and BCL-2 in consecutive sections of xenografted tumor tissues; scale bar: 50 *μ*m.

To investigate the role of ICT1 in tumor growth *in vivo*, we established a xenograft model by stably silencing ICT1 and transfecting BCL-2 into U-2OS cells. As shown in Figures 4(a)–4(d), the stable ICT1-knockdown group exhibited significantly decreased the xenografted tumor growth and a decreased tumor burden compared to the control group. Moreover, the compromised tumorigenic potential in the ICT1-knockdown group was partly offset by overexpression of BCL-2. TUNEL and immunohistochemical staining assays showed that revealed declining expression of Ki67 and a rising rate of apoptosis in the xenografted tumors of ICT1-knockdown group (Figure 4(e)). These data further indicate that ICT1 promoted OS cell growth targeting BCL-2. Finally, we confirmed that ICT1 protein levels in xenografted tumor tissues had a positive correlation with the expression levels of BCL-2 (Figure 4(f)). The above results revealed that ICT1 may promote growth of OS cells by targeting BCL-2 *in vivo*.

## 4. Discussion

Previous studies have found significant biological effects of ICT1 on the regulation of tumor growth and apoptosis. In hepatocellular carcinoma, ICT1 promoted cell growth by facilitating cell cycle progression and preventing apoptosis [23]. In non-small-cell lung cancer, depletion of ICT1 inhibited proliferation and promoted apoptosis [6]. A recent study revealed that ICT1 was upregulated in gastric cancer and promoted its invasion and migration [7]. However, the expression and biological effects of ICT1 in OS have not been studied. Here, we demonstrated that the levels of the ICT1 expression were markedly higher in the OS cell lines. Additionally, we showed that knockdown of ICT1 inhibited proliferation and promoted apoptosis of OS cells *in vitro* and *in vivo*.

The BCL-2 family of proteins, known as the apoptotic proteins, can be broadly divided into proapoptotic and antiapoptotic. BCL-2, which belongs to the antiapoptotic class, is a typical oncogene [24]. Since Fukuhara and Rowley discovered the BCL-2 protein, evidence has consistently demonstrated that BCL-2 plays an important role in cancer cell growth, metastasis, angiogenesis, and apoptosis [25]. Furthermore, recent studies have confirmed that some microRNAs can inhibit OS growth by regulating BCL-2 and that some drugs can promote OS apoptosis through BCL-2 [22, 26–28]. As the intersection of many carcinogenic signaling pathways, STAT3 is involved in cell proliferation. Furthermore, previous studies have revealed that STAT3 signaling is an important signaling molecule involved in many cellular processes, including inflammation, apoptosis, and cell-cycle control, and has become a promising target for cancer treatment [29–31]. Furthermore, Dziennis in 2007 showed that phosphorylated STAT3 promotes BCL-2 transcription [32]. A different study revealed that FZKA induced lung cancer cell apoptosis involving the STAT3/BCL-2/Caspase-3 pathway [33]. In our study, we showed that knockdown of ICT1 can alter the expression of apoptosis-related genes including BCL-2, BAX, cleaved caspase-3, and cleaved caspase-9 in OS cell lines. More importantly, we confirmed that ICT1 protein levels had a positive correlation with the expression levels of BCL-2 in both xenografted tumor tissues and tissues derived from OS patients and that ICT1 regulated BCL-2 by controlling STAT3 phosphorylation. The overexpression of BCL-2 also partly reversed the inhibitory effects of ICT1 knockdown on the growth of OS cell lines. However, the mechanisms by which ICT1 regulates the activity of STAT3 remain unknown. This question will be further explored in future studies.

In conclusion, our data confirmed that the ICT1 expression was increased in OS cells and that ICT1 promoted OS cell growth both *in vitro* and *in vivo* by inhibiting apoptosis through the STAT3/BCL-2 pathway. This study provides biological insights into the evolution of OS and suggests that ICT1 can be regarded as a new target for OS treatment in the clinic.

## Data Availability

The data used to support the findings of this study are included within the article.

## Supplementary Materials

Supplementary Materials Figure 1: (a) Knockdown efficacy of ICT1 in normal osteoblast cell line (hFOB1.19) was determined by western blotting. (b) ICT1 knockdown had little effect on the growth of the normal human osteoblast cell line (hFOB1.19) using colony formation assay.
