# Supplementary material for: ICT1 Promotes Osteosarcoma Cell Proliferation and Inhibits Apoptosis via STAT3/BCL-2 Pathway
Source: Biomed Res Int. 2021 Jan 22;2021:8971728. doi: 10.1155/2021/8971728 (PMC7853870; doi:10.1155/2021/8971728)
Supplement: Supplementary Materials — Figure 1: (a) Knockdown efficacy of ICT1 in normal osteoblast cell line (hFOB1.19) was determined by western blotting. (b) ICT1 knockdown had little effect on the growth of the normal human osteoblast cell line (hFOB1.19) using colony formation assay. [file 8971728.f1.docx]

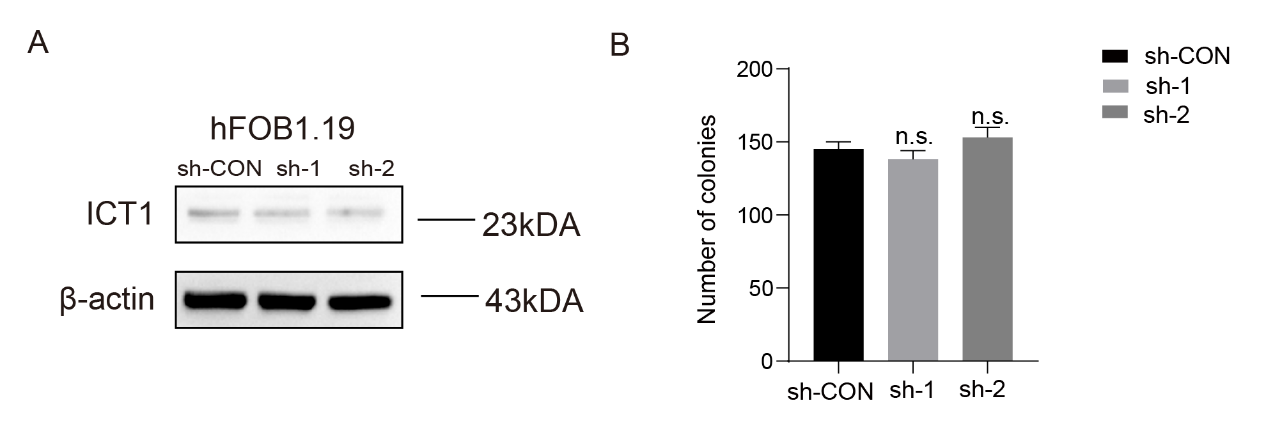
Figure 1 A. Knockdown efficacy of ICT1 in normal osteoblast cell line (hFOB1.19) was determined by western blotting. B. ICT1 knockdown had little effect on the growth of the normal human osteoblast cell line (hFOB1.19) using colony formation assay.
